# Application of Ionic Liquid-Based Ultrasonic-Assisted Extraction of Flavonoids from Bamboo Leaves

**DOI:** 10.3390/molecules23092309

**Published:** 2018-09-10

**Authors:** Liling Wang, Minge Bai, Yuchuan Qin, Bentong Liu, Yanbin Wang, Yifeng Zhou

**Affiliations:** 1 Zhejiang Academy of Forestry, Hangzhou 310023, China; echo22239@163.com (L.W.); mingebai1966@126.com (M.B.); hzqinchuan@126.com (Y.Q.); echo22239@sina.com (B.L.); 2 School of Biological and Chemical Engineering, Zhejiang University of Science and Technology, Hangzhou 310023, China; 3 Zhejiang Provincial Key Lab for Chemical and Biological Processing Technology of Farm Produces, Hangzhou 310023, China; 4 Zhejiang Province Collaborative Innovation Center of Agricultural Biological Resources Biochemical Manufacuring, Hangzhou 310023, China

**Keywords:** *Phyllostachys heterocycle*, bamboo leaves, ionic liquid, ultrasonic-assisted extraction, flavonoids

## Abstract

Ionic liquids (ILs), known as environmentally benign “green” solvents, were developed as an optimal solvent for the green extraction and separation field. In this paper, an ionic liquid-based ultrasonic-assisted extraction (IL-UAE) of flavonoids (FVs) from bamboo leaves of *Phyllostachys heterocycla* was developed for the first time. First, 1-butyl-3-methylimidazolium bromide ([Bmim] Br), with the best extraction efficiency, was selected from fifteen ionic liquids with diverse structure, like carbon chains or anions. Then, the influencing parameters of ionic liquid (IL) concentration, liquid-solid ratio, ultrasonic time, and ultrasonic power, were investigated by single factor tests, and further optimized using response surface methodology (RSM). In the optimization experiment, the best conditions were 1.5 mol/L [BMIM]Br aqueous solution, liquid-solid ratio 41 mL/g, ultrasonic time 90 min, and ultrasonic power 300 W. Furthermore, the microstructures of bamboo leaves and the recovery of FVs and [BMIM]Br were also studied. Therefore, this simple, green and effective IL-UAE method has potentiality for the extraction of FVs from bamboo leaves for the large-scale operations.

## 1. Introduction

Bamboo, a tropical and subtropical plant, which belongs to the Gramineae family and Bambuseae subfamily, has high edible and economic value. China, known as the “bamboo kingdom”, is the largest edible bamboo-producing country and has about 400 species in 44 genera, occupying 3% of global forest area, mainly distributed in the Yangtze river basin and south of the Yangtze river, which has large substantial reserves and potential economic value. The leaves of bamboo have a long history of edible and medicinal use in the vast areas of China and even Southeast Asia. In 1998, the Chinese ministry of health determined that it was a medicinal herb, so the leaves themselves were safe.

**Figure 1:**
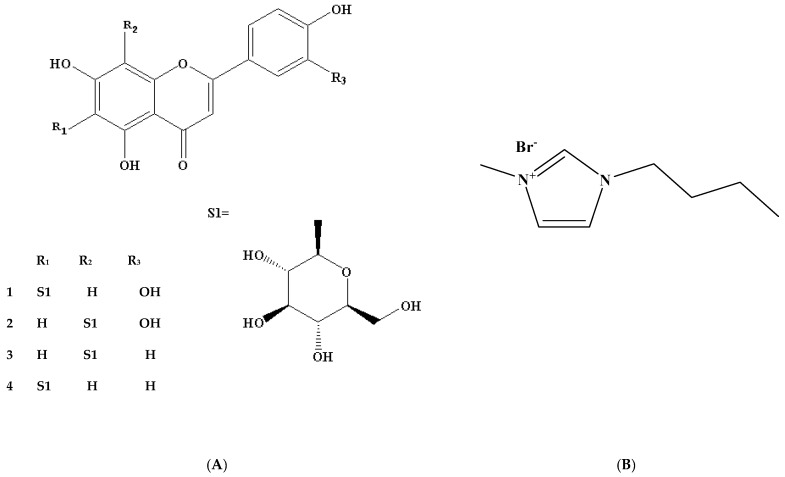
Chemical structures of flavonoids (FVs) from bamboo leaves (**A**): isoorientin (**1**), orientin (**2**), vitexin (**3**), isovitexin (**4**), and 1-butyl-3-methylimidazolium bromide ([Bmim] Br) (**B**).

Previous studies show that bamboo leaves contain flavonoids (FVs), lactones, phenolic acids, polysaccharides, amino acids and other substances beneficial to the human body. FVs are a type of important antioxidant ingredient from bamboo leaves (AOB), which have been approved as a natural food additive by the Chinese Ministry of Health. They can be added to aquatic products, meat products, puffed foods, cereals, and more [1,2]. Moreover, FVs, mainly including orientin, homoorientin, vitexin, and isovitexin (Figure 1A), in particular, are a type of important antioxidant, involved in such processes as anti-aging, antitumor [3,4], scavenging free radicals [5], enhancing immunity [6], blocking nitrosation [7], adjusting blood fat [8,9] and blood sugar [10], and more.

At present, the common bamboo leaf FVs extraction methods include solvent extraction, ultrasonic extraction method, and enzymatic methods [11,12]. Generally, traditional solvent extraction is low cost, uses simple equipment, but results in a lower extraction yield. On the other hand, because of the polarity of solvents such as water, ethanol aqueous solubility is widespread, so the selectivity is not strong, and the extract impurities remain (such as proteins, polysaccharides, and tannins, for example). Although the extraction rate of the ultrasonic method is better than that of hot reflux, the subsequent separation is also more complicated. Enzymatic methods can improve the extraction rate of FVs, but the enzymes are volatile and expensive. Based on these shortcomings, researchers worked on establishing a miniaturized extraction method with low solvent consumption, fast extraction speed, and simplicity and high efficiency. Herein, the use of ionic liquids (ILs) has received attention, at present, for their good performance in all aspects. Early on, ionic liquid solutions, as solvents, were successfully applied in the microwave-assisted extraction (MAE) of polyphenolic compounds from medicinal plants, like polyphenolic compounds from *Psidium guajava* Linn. (*P. guajava*) leaves and *Smilax china* (*S. china*) tubers [13], and three alkaloids *N*-nornuciferine, O-nornuciferine, and nuciferine from lotus leaf [14].

ILs as a kind of novel solvent in the extraction and/or purification of bioactive compounds, such as phenolic acids [15,16], alkaloids [17], fats [18], essential oils [19], carotenoids [20], vitamins [21], amino acids [22], among others, to more complex molecules, such as nucleic acids [23], proteins [24], enzymes [25], and antibodies [26], have been successfully applied. As a green solvent, ILs are liquid molten salts below 100 °C, and typically consist of large and unsymmetrical organic cations and organic or inorganic anions. Moreover, ILs have a large number of ion combinations, and the possibility of designing task-specific fluids as tunable designer solvents. Currently, much attention is given today, to the application of ILs in the fields of natural bioactive products for their extraction and separation, together with hydrolysis. Moreover, ionic liquid-assisted extraction of FVs has been reported. For instance, 1-butyl-3-methylimidazolium tetrafluoroborate (0.4 mL, 250 mM) as the elution solvent was selected for the extraction of flavonoid glycosides from lime fruit [27]; Zhang and his workers [28] developed a novel technique, ionic liquid–water–organic solvent three phase microextraction (ILWOS-3p-ME), to simultaneously preconcentrate and determine FVs and anthraquinones in Chinese herbal formula and its preparations; ethyl acetate and 0.5 mol/L ionic liquid 1-butyl-3-methylimidazolium chloride aqueous solution was used to separate four FVs from *Rhodiola rosea* by on-line [29]; Wei and his coworkers used [BMIM]BF_4_ (1-butyl-3-methylimidazolium tetrafluoroborate) solution to perform ultrasound-assisted extraction of FVs from different parts of *Lysimachia clethroides* Duby [30]; More recently, 1-butyl-3-methylimidazolium tetrafluoroborate ([C4mim]BF_4_) was selected as the extraction solvent for the orientin and vitexin extraction [31]. Above all, it demonstrated that ionic liquids are promising new alternative solvents to conventional solvents in extraction process areas. In addition, ionic liquid-based ultrasonic-assisted extraction (IL-UAE) can improve the extraction efficiency from herbal medicines [32,33,34], and the stability of ILs was proved after the recovery experiments [35,36].

However, there are no reports on extraction methods of bamboo leaves using ionic liquids. In this paper, different ionic liquids were investigated, and 1-butyl-3-methylimidazolium bromide ([BMIM]Br) (Figure 1B) was selected for the extraction of FVs from bamboo leaves of *Phyllostachys heterocycla*. Then, the ionic liquid concentration, ultrasonication time, and liquid-solid ratio, were optimized for efficient extraction of FVs. Here high-performance liquid chromatography (HPLC) was used to quantify the contents of four FVs in bamboo leaves. Finally, we also studied the recovery of target products for yield tests and ionic liquid for reutilization.

## 2. Materials and Methods

### 2.1. Materials and Apparatus

All chemicals involved in this study were analytical reagent grade, if not otherwise stated. Acetonitrile was chromatographic grade for HPLC use, and purchased from Shanghai Aladdin Chemical Co. LTD., China, as was the case for all the ionic liquids (the purity is greater than or equal to 97%). Experimental water was prepared by Milli-Q3 water system (w ≥ 0.999, Millipore, Bedford, MA, USA). All standard compounds were purchased from Chengdu purechem-standard co., Ltd. Bamboo leaves of *Phyllostachys heterocycla* was collected from Anji country, Zhejiang province, China; and then the raw materials were dried, milled, and passed through a 60 mesh stainless steel sieve. All samples were stored in desiccators until use.

KQ-300DE ultrasonic extractor (Kunshan Ultrasonic Instruments Manufacture Co. Ltd., Jiangsu, China) were utilized in the extraction step. HPLC were performed on an e2695 separation system, including a quaternary pump, an autosampler and a column oven; FVs in bamboo leaves were analyzed by an Waters 2998 Photodiode Array Detector, and [Bmim] Br was determined by a Waters 2424 evaporative light-scattering detector (Waters Co., Milford, MA, USA). Morphology of herbal powders was observed with field-emission scanning electron microscope (Quanta 250 FEG, FEI, Hillsboro, OR, USA).

### 2.2. Quantitative Analysis of FVs

**Figure 2:**
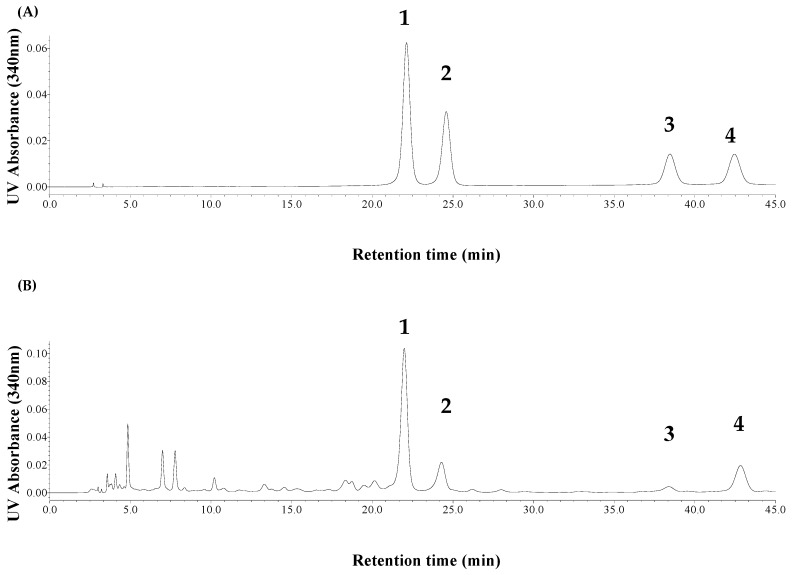
HPLC-UV chromatograms of FVs standards (**A**) and typical sample of bamboo leaves (**B**).

The HPLC analysis for FVs was carried out on an XBridge^TM^ C18 chromatographic column (4.6 mm × 250 mm, 5 μm.) at column temperature of 30 °C. The mobile phase was composed of 0.5% acetic acid–water solution and acetonitrile (*v*/*v*, 90:10), and the flow rate was 1.0 mL/min. The detection wavelength was set at 340 nm, and injection volume was 20 μL. The standard curves of four FVs were shown in Table 1, where y and x were its concentration (µg/mL) and the value of peak area, respectively. The results indicated the calibration curves showed good linear regression (*R*^2^ > 0.9990) within the test ranges. Moreover, the four FVs could be well separated in standards or samples with coexisting ionic liquid (see Figure 2). In this work, the extraction yield (%) of total FV contents was calculated by the content of four FVs, according to the analysis by HPLC.

### 2.3. Screening of ILs

Powders (0.5 g) of bamboo leaves and 10 mL aqueous solutions of the different ionic liquids (1 mol/L) were placed in Erlenmeyer flask, and then extracted with ultrasonication. Then, the extracting solution was filtered using a 0.45 μm microporous membrane, and methanol was added to a constant volume for HPLC analysis.

### 2.4. Optimization of IL-UAE by Single Factor Tests and Response Surface Methodology (RSM)

The single factor experiments were performed in several combinations, such as using different ionic liquid concentration (0, 0.25, 0.5, 0.75, 1, 1.25, and 1.5 mol/L), liquid-solid ratios (10, 20, 30, 40, 50, 60 g/mL), extraction times (10, 20, 30, 40, 50, 60, and 90 min), and ultrasonic power (120, 180, 240, 300 W) in the ultrasonic extractor. A Box–Behnken design was applied for RSM optimization with Design-Expert 8.0.6 Trail software (Stat-Ease, Minneapolis, MN, USA). Based on the above single factor experimental results, the chosen factor boundaries of ionic liquid concentration (A), liquid-solid ratio (B), and ultrasonic time (C) were listed in Table 2. The experiments were performed in random order to avoid systematic errors.

### 2.5. Recovery of Products and ILs

**Figure 3:**
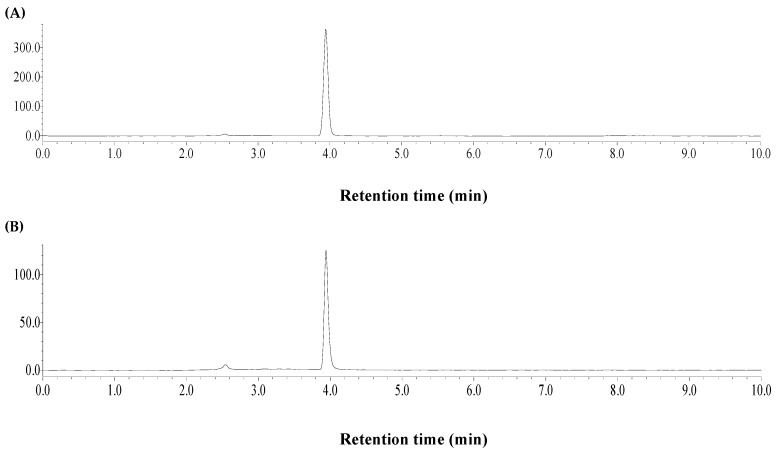
HPLC-ELSD chromatograms of [Bmim] Br standard (**A**) and recovery sample from bamboo leaves (**B**).

After extraction process, the extract was firstly diluted 3-fold by deionized water. Then, some insoluble organic solvents were tested for liquid–liquid extraction of FVs from IL. The recovery of FVs was determined according to the HPLC method in Section 2.2. The recovery performance of IL can be determined by HPLC-ELSD. The HPLC-ELSD detection of [Bmim] Br was performed using methanol-water (10:90, *v*/*v*) as the mobile phase, and 1.0 mL/min flow rate, 10 μL injection volume, 30 °C column temperature. The optimal ELSD conditions were nebulizing gas pressure, 25 psi; evaporator temperature, 60 °C; and drift tube temperature of 60% heat power (36 °C). Based on the above, the retention time of [Bmim]Br was 3.9 min (Figure 3). The calibration curve equation was Y = 1.8788 X + 5.7968 (*R*^2^ = 0.9997, *n* = 5), which suggested good linearity in the range 0.4148–3.3184 mg/mL, where Y and X were obtained by the logarithm of peak area and IL concentration (mg/mL), successively.

### 2.6. Conventional Reference Extraction Methods (RE)

Heat reflux extraction (HRE) with 80% ethanol at 80 °C (RE1), UAE with pure water (RE2), and ethanol (RE3) were selected as reference extraction methods for FVs from bamboo leaves. The optimum conditions (temperature, time, but solvent type excluded) were used for the following experiments. Sample powder (1.0 g) was added with 41 mL of each solution, and was extracted for 90 min. Then, HPLC analysis was carried out on the filtrate after filtering through a microporous membrane.

## 3. Results and Discussion

### 3.1. Screening of ILs for the Extraction of FVs

**Figure 4:**
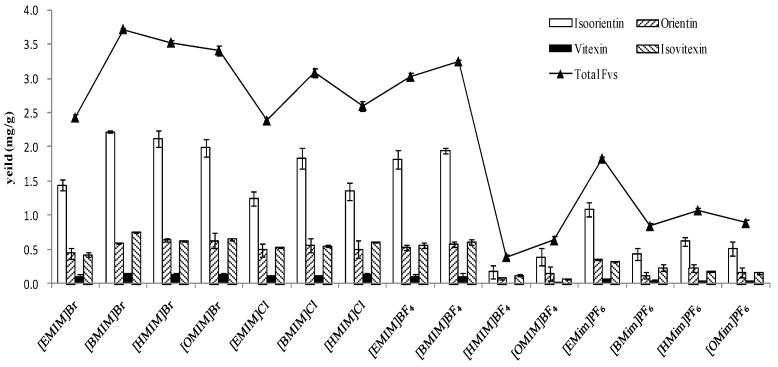
Performance comparison of cations and anions on the extraction efficiency of FVs.

For ILs, selecting the appropriate solvent is of great important to obtain a satisfactory extraction efficiency of the target product. High selectivity and good recovery for the target compounds were the key factors to be considered, which are mainly determined by the structure and physicochemical properties of ionic liquids. As shown in Figure 4, fifteen kinds of ILs, l-alkyl-3-methylimidazolium ionic liquids with different anions and cations, were investigated in IL-UAE process. Generally, we select the IL with high extraction of FVs and economic considerations at the same time. Based on the above, [Bmim][Br] was found to be the most ideal IL for extraction of FVs. Again, it demonstrated that the solubilities of FVs were strongly anion-dependent [37]. From Figure 4, for [Bmim]-based ionic liquids with Br^−^, Cl^−^, BF_4_^−^, and PF_6_^−^, the obtained extraction yields indicated that Br^−^ was the most efficient than the other three anions. The types of anions not only affect the density, viscosity, and solubility of ionic liquids, but also determine their interaction with the target components. For [Bmim]Br, there is actually a competitive process. On one hand, bromide ions in ionic liquids can form hydrogen bonds with water; on the other hand, they can also combine with the target FVs to form hydrogen bonds, which are stronger than that with water. Strong interactions can occur when IL molecules encounter target components in plant cells. Cations of [Bmim]Br provide π–π interactions with the parent nucleus of FVs. Similar results were observed in the extraction of puerarin from Radix *Puerariae lobatae* [32], and puerarin is also a kind of C glycoside flavonoid as orientin, homoorientin, vitexin, and isovitexin.

### 3.2. Effects of Various Conditions on the Extraction of FVs

**Figure 5:**
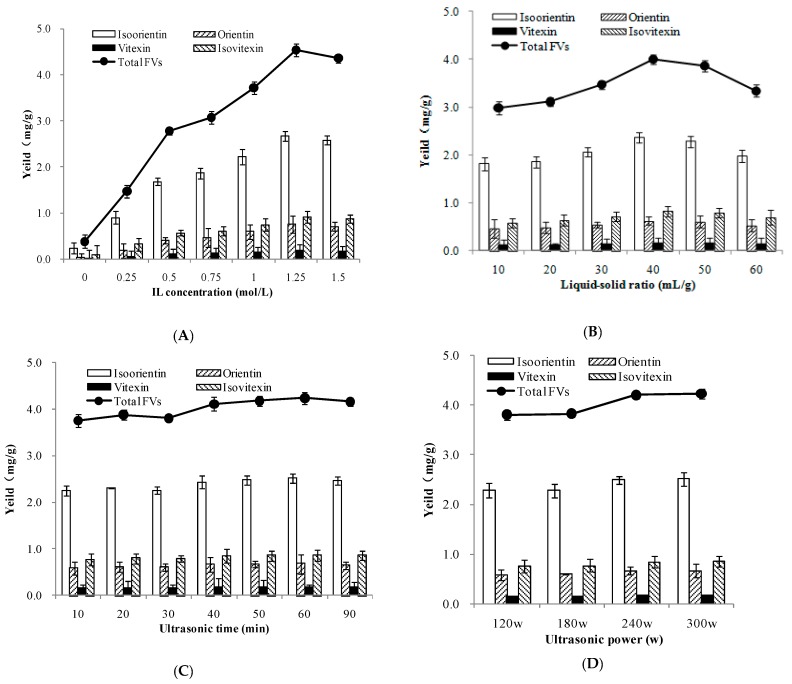
Effects of IL concentration (**A**), liquid-solid ratio (**B**), extraction time (**C**), and ultrasonic power (**D**) on the extraction efficiency of FVs.

Four influencing factors—the IL concentration, liquid-solid ratio, ultrasonic time, and ultrasonic power—were selected as the major factors to test the extraction yield in the study, according to the results of previous studies [32,33,34,35,36,37,38]. In order to maximize the yield, a series of different concentrations of IL were designed. The results in Figure 5A suggested, with the increase of ionic liquid concentration, the extraction yield of isoorientin and total FVs showed an upward trend. A maximum extraction yield was obtained when the IL concentration was 1.25 mol/L. The overall trend of the extraction rate of total FVs showed that the extraction rate was the most significant in 0–1.25 mol/L ionic liquid, and extraction yield of total FVs became higher, and mainly followed the isoorientin increase. From 1.25 to 1.5 mol/L, the extraction amount of total FVs began to decrease gradually following the isoorientin decrease. Considering better optimization for other factors, a [Bmim]Br concentration range of 1 mol/L was selected for subsequent single factor experiment; the IL concentration range of 0.5–1.5 mol/L was adopted for RSM optimization.

Besides the concentrations of ionic liquids, the liquid-solid ratio also plays an important role in the extraction efficiency. If the liquid-solid ratio is too small, incomplete extraction of target product will happen, because insufficient solvent prevents the extractant from dissolving; on the contrary, too large of a liquid-solid ratio will lead to the increase of solution viscosity and other nontarget products, and it will not be convenient for the extraction transfer process and recovery of ILs. To maximize the yield, experiments were carried out with a gradient of liquid-solid ratios (10, 20, 30, 40, 50, and 60 mL/g). From Figure 5B, the product yields increased significantly from 20 to 40 mL/g, and when the ratio was 40 mL/g, the extraction rate of four compounds reached the highest, and then the extraction rate decreased with the continuous increasing of the liquid-solid ratios. The results indicated that the liquid-solid ratio of 40 mL/g is enough for complete extraction of total FVs used in the following experiments.

Ultrasonic time is regarded as an important factor affecting product yield. As shown in Figure 5C, the slight change in extraction yields extended from 50 to 90 min. This phenomenon implied that FVs were stable in the extraction process, so 30–90 min was suitable for further RSM optimization.

In addition, ultrasonication power was evaluated at 120, 180, 240, and 300 W (Figure 5D). It was found the extraction yield of total FVs was apparently increased when the ultrasonic power increased, and the optimum ultrasonic power was 300 W. Therefore, the ultrasonic power was fixed at 300W in RSM optimization.

### 3.3. Optimization by RSM 

The different extraction yields of total FVs from 2.193 to 4.592 mg/g were obtained according to RSM optimization experiments (see in Table 3). Furthermore, the curve regression equation was given as follows:

Y (%) _FVS_ = 4.00 + 0.71 × A+0.12 × B+0.27 × C − 0.30 × AB − 0.097 × AC + 0.21 × BC − 0.32 × A^²^ − 0.33 × B^²^ + 0.034 × C^²^,

where Y (%) was the extraction efficiency of total FVs from bamboo leaves, and A, B, and C were the coded values of the factors of the ionic liquid concentration (A), liquid-solid ratio (B), and ultrasonic time (C), respectively. The coefficient *R*^2^ = 0.9954 implied excellent fitting correlation between the model and the experiment values.

As shown in Table 4, the value results of model (*p* < 0.0001) and ‘‘Lack of Fit’’ (*p* > 0.05) further indicated that the regression model was effective for predicting the experiment results. The ionic liquid concentration (A), liquid-solid ratio (B), and ultrasonic time (C) were significant factors (*p* < 0.01), which was also verified by single factors tests.

**Figure 6:**
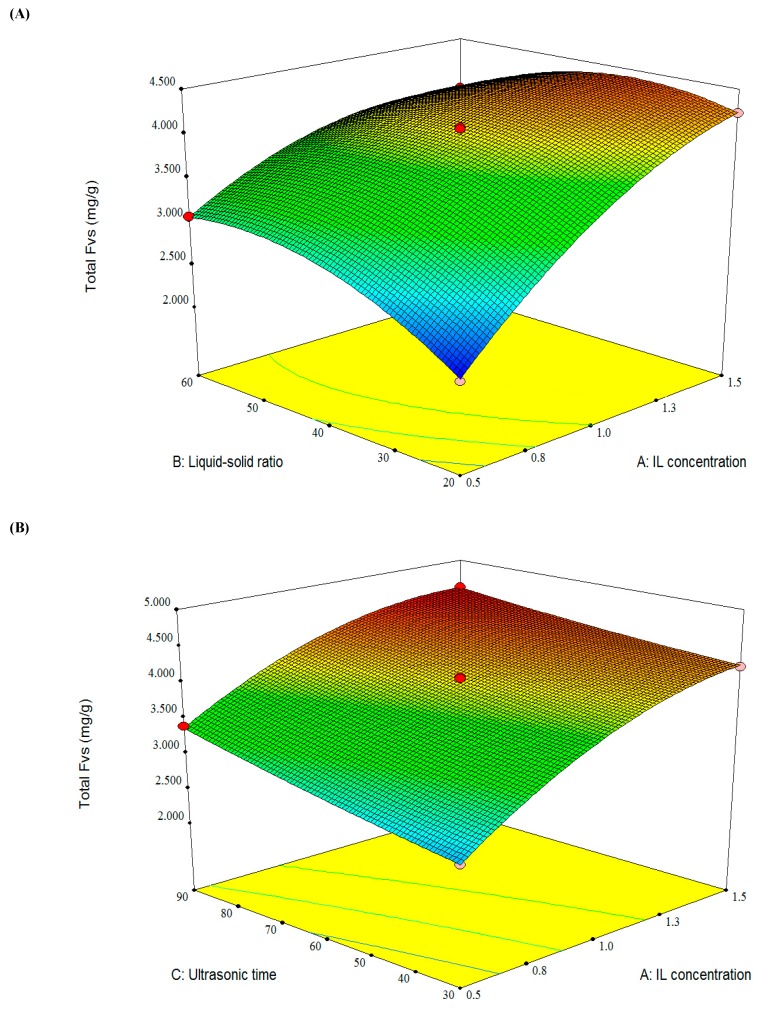

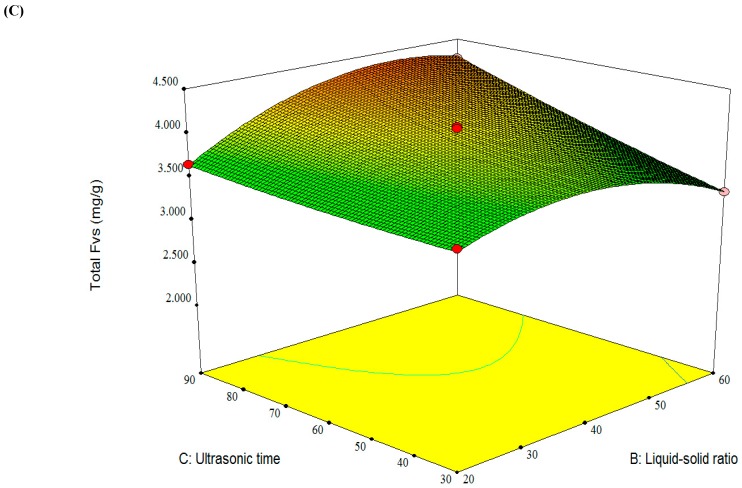
Response surface plots showing the effects of variables on the total FVs: (**A**) interaction of the IL concentration and liquid-solid ratio; (**B**) interaction of the ionic liquid (IL) concentration and ultrasonic time; and (**C**) interaction of the liquid-solid ratio and ultrasonic time.

As shown in Figure 6, the three-dimensional plots reflected the interactive effects of the three pairs of variables on the total FVs, and a maximum point depicted in three surface diagrams. It can be found that the total FVs increased with the increasing of the ionic liquid concentration (A), and ultrasonic time (C), and a slope curve of total FVs was affected by liquid-solid ratio (B). The highest extraction yield of total FVs could be achieved by predicted conditions as follows, the ionic liquid concentration (A) of 1.5 mol/L, liquid-solid ratio (B) of 41 mL/g, and ultrasonic time (C) of 90 min. Under these predicted conditions, the average total FVs yield from bamboo leaves by IL-UAE was 4.592 mg/g (*n* = 3), which is very close to the predicted value of 4.591 mg/g.

**Figure 7:**
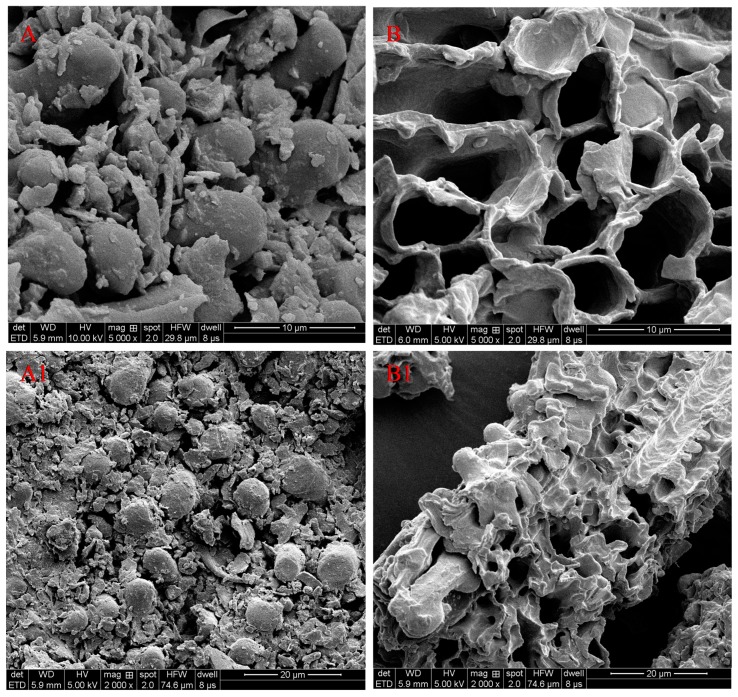
SEM photos of bamboo leave particles before extraction (**A**,**A1**), after extraction with [Bmim]Br (**B**,**B1**).

Moreover, the morphological changes before and after being extracted by IL aqueous solution were examined by scanning electron microscope (SEM, see Figure 7). From the picture, before extraction (Figure 7A,A1), the round particles were distributed on the surface of the bamboo leaves sample and there were no holes. However, after the process of IL-UAE extraction, the bamboo leaves had no complete cell structure, and the cell walls were almost destroyed (Figure 7B,B1), which accelerated the dissolution rate of FVs. This is because ultrasonic waves have strong penetration, which can make water molecules rotate violently inside and outside the cell wall, and increase the cell wall penetrability and temperature in the cell. After that, the pressure in the cell increases, exceeding the capacity of the cell wall itself. Finally, the cell wall breaks down and FVs are released in large quantities.

### 3.4. Recovery of Products and ILs

In a previous study, water-immiscible organic solvents, like *n*-butanol and chloroform, were the ideal solvents to recover products and ILs [16]. In this process, FVs were separated from an ionic liquid solution by an organic solvent. After extraction three times (Vextract: Vorganic solvent = 1:1 in each time), IL and FV recovery (%) can be calculated, according to their concentrations before and after extraction by HPLC analysis. Only *n*-butanol was found to have good recovery of both FVs and ILs (see in Table 5). A large majority of FVs (97.87 ± 0.25%) and ILs (96.52 ± 0.63%) were recovered from the extract.

### 3.5. Comparison with Traditional Methods

**Figure 8:**
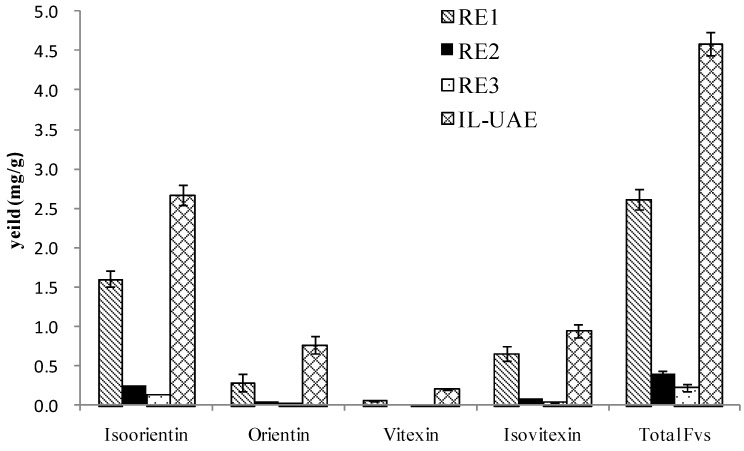
Comparison of ionic liquid-based ultrasonic-assisted extraction (IL-UAE) with reference extraction methods (RE1-3).

In the end, a comparison was performed with the conventional UAE and HRE method. The results are summarized in Figure 8. The extraction yield of total FVs by IL-UAE was demonstrated to be much higher than other reference extraction methods. It suggested that IL-UAE was an effective procedure for FV extraction from bamboo leaves, and that the IL aqueous solution played a critical role in this process.

## 4. Conclusions

In this work, an efficient IL-UAE method was developed for extraction of FVs from bamboo leaves. Five IL-UAE parameters, including the type of IL, IL concentration, liquid-solid ratio, and ultrasonic power and time, were optimized by single-factor experiments. After optimization by RSM, the highest yield was achieved by using 1.5 mol/L [BMIM]Br aqueous solution, liquid-solid ratio of 41 mL/g, ultrasonic time of 90 min, and 300 W ultrasonic power. In addition, it was found that *n*-butanol extraction had the ideal separation performance for [BMIM]Br ionic liquid and FVs. Compared with other reference methods, IL-UAE is an environmentally friendly method with higher yield, which could be also an alternative method to extract the FVs from bamboo leaves.

## Figures and Tables

**Table 1:** Quantitative curves for the analytes in bamboo leaves.

No.	Compound	Linear Equation	Linear Range (μg/mL)	*R* ^2^
1	Isoorientin	Y = 33,927x + 69,381	8.240~206.000	0.9996
2	Orientin	Y = 28,916x + 3955.1	2.500~62.500	0.9998
3	Vitexin	Y = 29,732x − 7953.1	1.744~17.440	0.9992
4	Isovitexin	Y = 34,931x − 10515	2.856~71.400	0.9994

**Table 2:** The actual parameters of the three factors-three levels in the Box-Behnken experiment.

Level	Factor A: IL Concentration (mol/L)	Factor B: Liquid-Solid Ratio (mL/g)	Factor C: Ultrasonic Time (min)
-1	0.5	20	30
0	1	40	60
1	1.5	60	90

**Table 3:** Box-Behnken experiment design and results.

Run.	A	B	C	Total Flavonoids (FVs) (mg/g)
1	0	0	0	4.081
2	−1	0	−1	2.639
3	1	−1	0	4.233
4	0	−1	−1	3.557
5	−1	−1	0	2.193
6	0	1	1	4.263
7	0	0	0	3.973
8	−1	1	0	3.067
9	0	−1	1	3.652
10	0	0	0	3.996
11	0	0	0	3.887
12	−1	0	1	3.396
13	1	1	0	3.894
14	1	0	1	4.592
15	0	0	0	4.062
16	1	0	−1	4.224
17	0	1	−1	3.340

**Table 4:** Analysis of variance (ANOVA) for the response surface model of total FVs from bamboo leaves.

Source	Sum of Squares	df	Mean Square	*F*-Value	*p*-Value
Model	6.19	9	0.69	167.24	<0.0001
A	3.99	1	3.99	968.84	<0.0001
B	0.11	1	0.11	26.26	0.0014
C	0.57	1	0.57	139.63	<0.0001
AB	0.37	1	0.37	89.41	<0.0001
AC	0.038	1	0.038	9.19	0.0191
BC	0.17	1	0.17	41.57	0.0004
A^2^	0.44	1	0.44	105.93	<0.0001
B^2^	0.46	1	0.46	112.24	<0.0001
C^2^	4.991 × 10^−3^	1	4.991 × 10^−3^	1.21	0.3072
Residual	0.029	7	4.115 × 10^−3^		
Lack of Fit	4.849 × 10^−3^	3	1.616 × 10^−3^	0.27	0.8448
Pure Error	0.024	4	5.989 × 10^−3^		
Cor Total	6.22	16			

**Table 5:** Results of recovery of total FVs and ionic liquids (ILs) with organic solvents (%) (*n* = 3).

	Ethyl Acetate	Chloroform	*n*-Butanol
**Total FVs**	20.16 ± 0.33	87.20 ± 0.16	97.87 ± 0.25
**ILs**	98.65 ± 0.51	97.65 ± 0.35	96.52 ± 0.63
